# Author Correction to: Association of Bacteroides acidifaciens relative abundance with high-fibre diet-associated radiosensitisation

**DOI:** 10.1186/s12915-021-01066-5

**Published:** 2021-07-09

**Authors:** Chee Kin Then, Salome Paillas, Xuedan Wang, Alix Hampson, Anne E. Kiltie

**Affiliations:** 1 CRUK/MRC Oxford Institute for Radiation Oncology, Department of Oncology, University of Oxford, Old Road Campus Research Building, Off Roosevelt Drive, Oxford, OX3 7DQ UK; 2 Department of Zoology, University of Oxford, Oxford, UK; 3 Department of Biochemistry, University of Oxford, Oxford, UK

**Author Correction to: BMC Biol 18, 102 (2021)**

**https://doi.org/10.1186/s12915-020-00836-x**

Following publication of the original article [1], it has been brought to the authors’ attention that after 16S sequencing of the v1-8 region, the bacterium which we originally believed to be *F. prausnitzii* (a butyrate-producer) in our penultimate figure, Fig. 5k and Additional file 1: Figure S5B, was in fact *L. plantarum* (a lactate-producer). This does not affect the other data, especially the animal work, nor does it alter the final conclusions of this manuscript.

The correct Figs. 5 and S5 and their caption have been included below and the fully corrected version of Additional file 1 is attached to this Author Correction, and the original article [1] has been corrected.

**Fig. 5:**
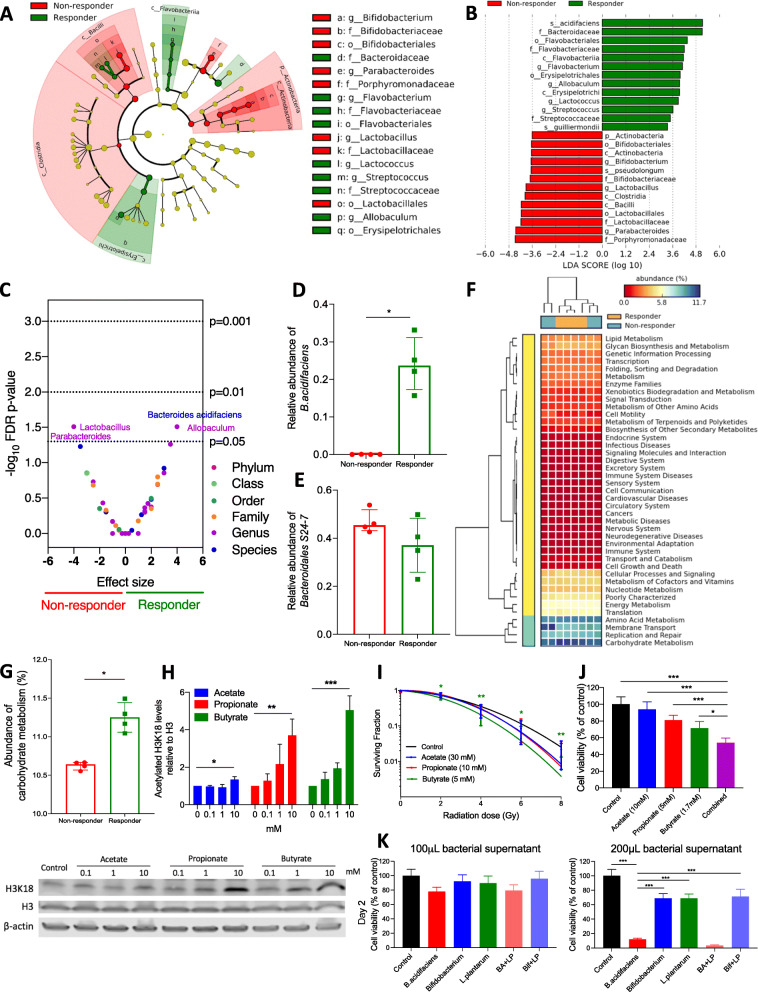
Differences in composition of the gut microbiome between responders and non-responders. **a** Taxonomic cladogram from LEfSe showing differences among taxa between responders and non-responders in the soluble HF group. Dot size is proportional to the abundance of the taxon. **b** Linear discriminant analysis (LDA) scores computed for differentially abundant taxa in the microbiomes of responders (green) and non-responders (red). Length indicates the effect size associated with a taxon, *p* = 0.05 for the Kruskal-Wallis test. **c** Discrete false-discovery rate of different abundant taxa in responders and non-responders in the soluble HF group. Differential abundance within all taxonomic levels in responders versus non-responders by Mann-Whitney *U* test. Dots are overlapping between *Bacteroides acidifaciens* and *Allobaculum*, and between *Lactobacillus* and *Parabacteroides*. Relative abundance of **d** *B. acidifaciens* and **e** *Bacteroidales S24-7* and in responders and non-responders in the soluble HF group. **f**, **g** Metagenomic functional prediction by PICRUSt of the gut microbiome in responders (*n* = 4) and non-responders (*n* = 4) in the soluble HF group with reference to the KEGG database level 2. Columns represent mice (responders, orange; non-responders, blue), and rows represent enrichment of predicted KEGG pathways (red, low enrichment; yellow, medium enrichment; blue, high enrichment). **h** Western blot analysis of histone acetylation levels of RT112 cells treated with SCFAs (*N* = 3). **i** Linear quadratic survival curves of IC10-treated RT112 cells with receiving irradiation of 0–8 Gy (*N* = 3). **j** Cell survival analysis of RT112 cells treated with single SCFA and combined SCFAs mixture (*N* = 3). Combined (purple bar) denotes SCFA mixture of 10 mM acetate, 5 mM propionate, and 1.7 mM butyrate **k** Reduced cell survival of RT112 cells by bacterial supernatants at day 2 (N=1). *BA*+*LP* denotes the cross-feeding of *B. acidifaciens* and *L. plantarum*, while *Bif*+*LP* denotes the cross-feeding of *Bifidobacterium* and *L. plantarum*. *p < 0.05; **p < 0.01; ***p < 0.001

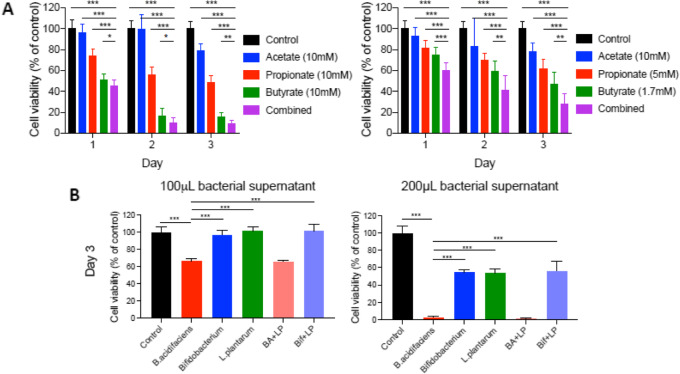



**Figure S5.** Cell survival analysis of RT112 bladder tumour cells treated with SCFAs and bacterial supernatants. (A) Inhibition of cell viability of RT112 cells single SCFA and combined SCFAs mixture in a time-dependent manner (N=3). The combined SCFAs denote the mixtures of 10 mM butyrate, 10 mM propionate, 10 mM butyrate for the left-hand graph and the mixtures of 10 mM butyrate, 5 mM propionate, 1.7 mM butyrate for the right-hand graph. (B) Reduced cell survival of RT112 cells by bacterial supernatants at day 3 (N=1). *BA*+*LP* denotes the cross-feeding of *B. acidifaciens* and *L. plantarum*, while *Bif*+*LP* denotes the cross-feeding of *Bifidobacterium* and *L. plantarum.* *P<0.05; **P<0.01; ***P<0.001.

Furthermore, some sentences in the original article’s main text need to be corrected. The affected text has been highlighted in **bold typeface**, and the original article [1] has been corrected.

**Previous version**

Results

To validate the anti-tumoural effects of *B. acidifaciens*, we treated the bladder tumour cells with bacterial supernatants of *B. acidifaciens* and its cross-feeding with ***F. prausnitzii***, and compared their effects with *Bifidobacterium* (acetate-producer) and ***F. prausnitzii*** **(butyrate-producer)**. Bacterial supernatants of *B*. *acidifaciens* and its cross-feeding with ***F. prausnitzii*** significantly increased cytotoxicity of bladder tumour cells compared to the other supernatants in day 2 (Figure 5K) and in day 3 (Additional file 1: Figure S5B).

Discussion

In this study, we revealed that bacterial supernatant from *B. acidifaciens* and its cross-feeding with ***F. prausnitzii*** caused significantly higher levels of cytotoxicity compared to the other supernatants (Figure 5K and Additional file 1: Figure S5B). This result supports our finding that *B. acidifaciens* may drive the radiosensitising effect. Moreover, *B. acidifaciens in vitro* has a greater effect on cell kill than ***F. prausnitzii*** **(butyrate-producer**; p<0.001), implying that metabolites other than **butyrate** may be involved in its effect.

Methods

All bacterial strains were obtained from DSMZ-German collection of microorganisms. Three strains of bacteria, namely *B. acidifaciens* (*BA*; DSM 15896), *Bifidobacterium animali*s (*Bif*; DSM10140), ***F. prausnitzii*** **(*FP*; DSM17677)**, and two cross-feeding combinations (***BA+FP*** and ***Bif+FP***) were cultured in Gifu Anaerobic Broth, Modified (GAM; Nissui Pharmaceutical, Japan).

**Corrected version**

Results

To validate the anti-tumoural effects of *B. acidifaciens*, we treated the bladder tumour cells with bacterial supernatants of *B. acidifaciens* and its cross-feeding with ***L. plantarum***, and compared their effects with *Bifidobacterium* (acetate-producer) and ***L. plantarum*** **(lactate-producer)**. Bacterial supernatants of *B*. *acidifaciens* and its cross-feeding with ***L. plantarum*** significantly increased cytotoxicity of bladder tumour cells compared to the other supernatants in day 2 (Figure 5K) and in day 3 (Additional file 1: Figure S5B).

Discussion

In this study, we revealed that bacterial supernatant from *B. acidifaciens* and its cross-feeding with ***L. plantarum*** caused significantly higher levels of cytotoxicity compared to the other supernatants (Figure 5K and Additional file 1: Figure S5B). This result supports our finding that *B. acidifaciens* may drive the radiosensitising effect. Moreover, *B. acidifaciens in vitro* has a greater effect on cell kill than ***L. plantarum*** **(lactate-producer**; p<0.001), implying that metabolites other than **lactate** may be involved in its effect.

Methods

All bacterial strains were obtained from DSMZ-German collection of microorganisms. Three strains of bacteria, namely *B. acidifaciens* (*BA*; DSM 15896), *Bifidobacterium animali*s (*Bif*; DSM10140), ***L. plantarum*** **(*LP*)**, and two cross-feeding combinations (***BA+LP*** and ***Bif+LP***) were cultured in Gifu Anaerobic Broth, Modified (GAM; Nissui Pharmaceutical, Japan).

## Supplementary Information

**Additional file 1: Figure S1.** Similar bacterial components in the faecal and caecal microbiomes. **Figure S2.** Faecal butyrate levels and time taken for tumours to reach 50 mm^**3**^. **Figure S3.** Differences in composition of the gut microbiome when tumours reached 350 mm^**3**^. **Figure S4.** Individual mouse tumour growth curves. **Figure S5.** Cell survival analysis of RT112 bladder tumour cells treated with SCFAs and bacterial supernatants. **Figure S6.** Correlation of time to culling with *B. acidifaciens* or *Parabacteroides* genus abundance different groups. **Figure S7.** Effect of cage location of mice on relative abundance of *B. acidifaciens* and *Parabacteroides* genus. **Table S1.** Rodent diets used in the study with varying levels of cellulose or inulin per 4000 kcal. **Table S2.** Details mouse diets, cages, *B. acidifaciens* relative abundance and time of culling.
