# Supplementary material for: Author Correction to: Association of Bacteroides acidifaciens relative abundance with high-fibre diet-associated radiosensitisation
Source: BMC Biol. 2021 Jul 9;19:139. doi: 10.1186/s12915-021-01066-5 (PMC8272367; doi:10.1186/s12915-021-01066-5)
Supplement: Supplementary file 1 — Additional file 1: Figure S1. Similar bacterial components in the faecal and caecal microbiomes. Figure S2. Faecal butyrate levels and time taken for tumours to reach 50 mm3. Figure S3. Differences in composition of the gut microbiome when tumours reached 350 mm3. Figure S4. Individual mouse tumour growth curves. Figure S5. Cell survival analysis of RT112 bladder tumour cells treated with SCFAs and bacterial supernatants. Figure S6. Correlation of time to culling with B. acidifaciens or Parabacteroides genus abundance different groups. Figure S7. Effect of cage location of mice on relative abundance of B. acidifaciens and Parabacteroides genus. Table S1. Rodent diets used in the study with varying levels of cellulose or inulin per 4000 kcal. Table S2. Details mouse diets, cages, B. acidifaciens relative abundance and time of culling. [file 12915_2021_1066_MOESM1_ESM.docx]

**Additional file 1**

**
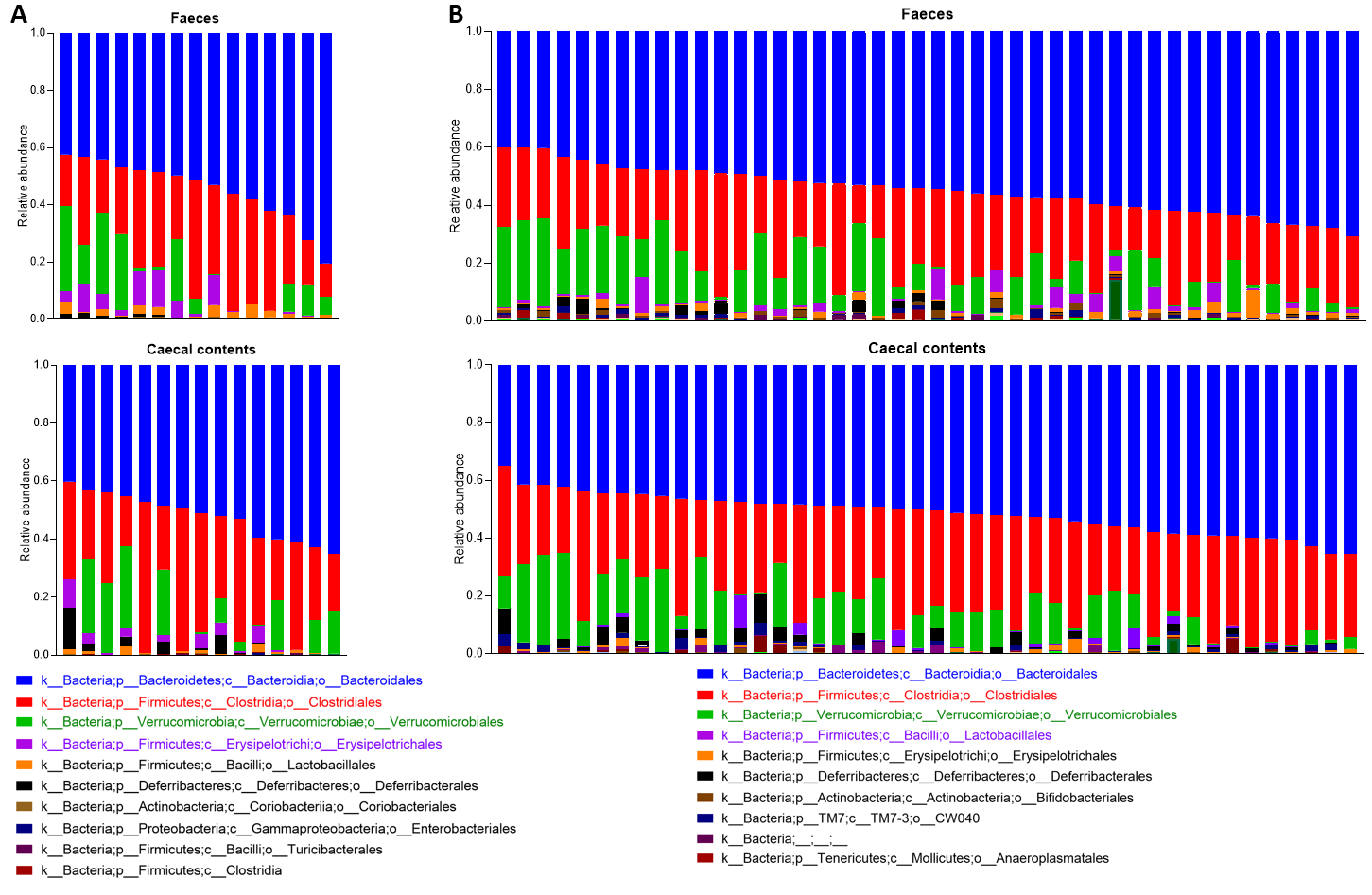
**

**Figure S1. Similar bacterial components in the faecal and caecal microbiomes.** Phylogenetic composition of faecal and caecal microbiomes at the order level when tumours reached (A) 50 mm^3^ (n = 15) and (B) 350 mm^3^ (n = 44). Samples were collected from faeces and caecal contents, and sorted by *Bacteroidales* proportion.


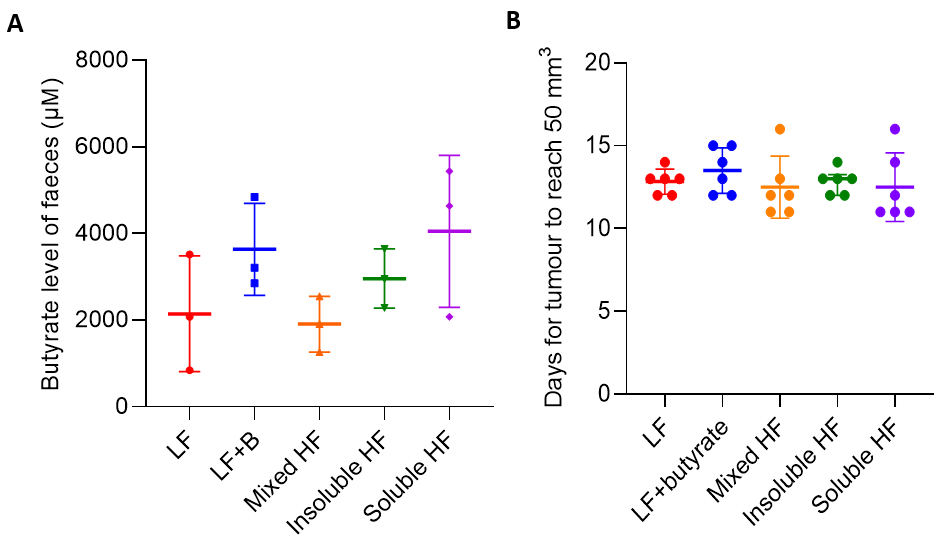


Figure S2. Faecal butyrate levels and time taken for tumours to reach 50 mm^3^. (A) Butyrate levels in the faeces at the time of culling. (B) All mice were culled when tumours reached 50 mm^3^, between 11 to 16 days after tumour inoculation, mean 12.8 (SD ± 1.4) days.


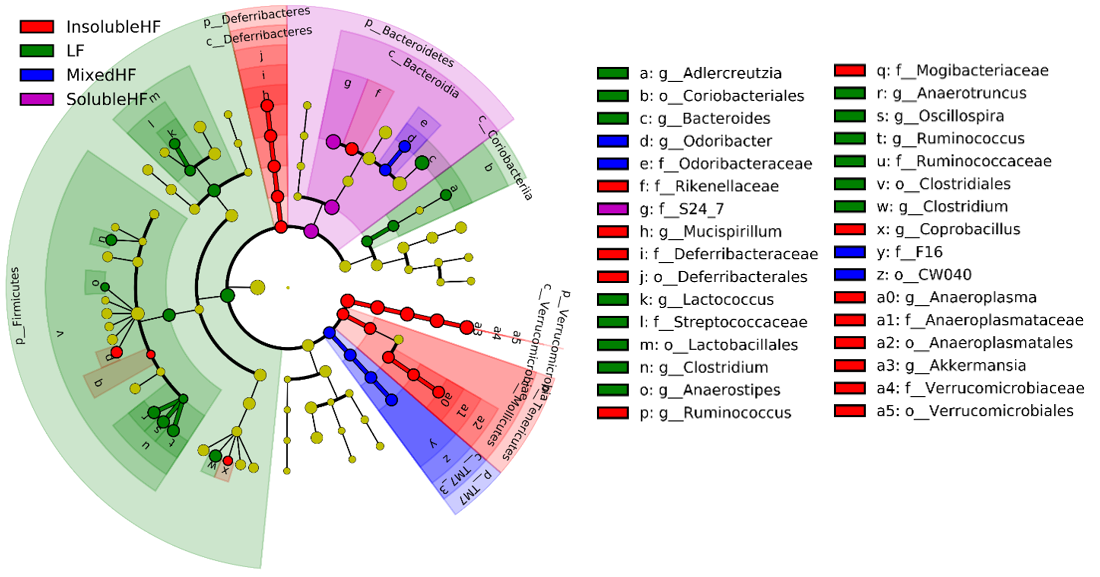
**Figure S3. Differences in composition of the gut microbiome when tumours reached 350 mm^3^.** Taxonomic cladogram from LEfSe showing differences in bacterial taxa at the genus level in the IR cohort when the tumours reached 350 mm^3^. The low fibre diet increased *Adlercreutzia, Coriobacteriales, Bacteroides, Lactococcus, Streptococcaceae, Lactobacillales, Oscillospira, Ruminococcus, Ruminococcaceae, Clostridiales, Clostridium*, the high mixed fibre increasing *Odoribacter, Odoribacteraceae, F16, CW040*, the high insoluble fibre diet increased *Rikenellaceae, Mucispirillum, Deferribacteraceae, Deferribacterales, Ruminococcus, Mogibacteriaceae, Coprobacillus, Anaeroplasma, Anaeroplasmataceae, Anaeroplasmatales, Akkermansia, Verrucomicrobiaceae, Verrucomicrobiales*, and the high soluble fibre diet increased *S24-7.*

**
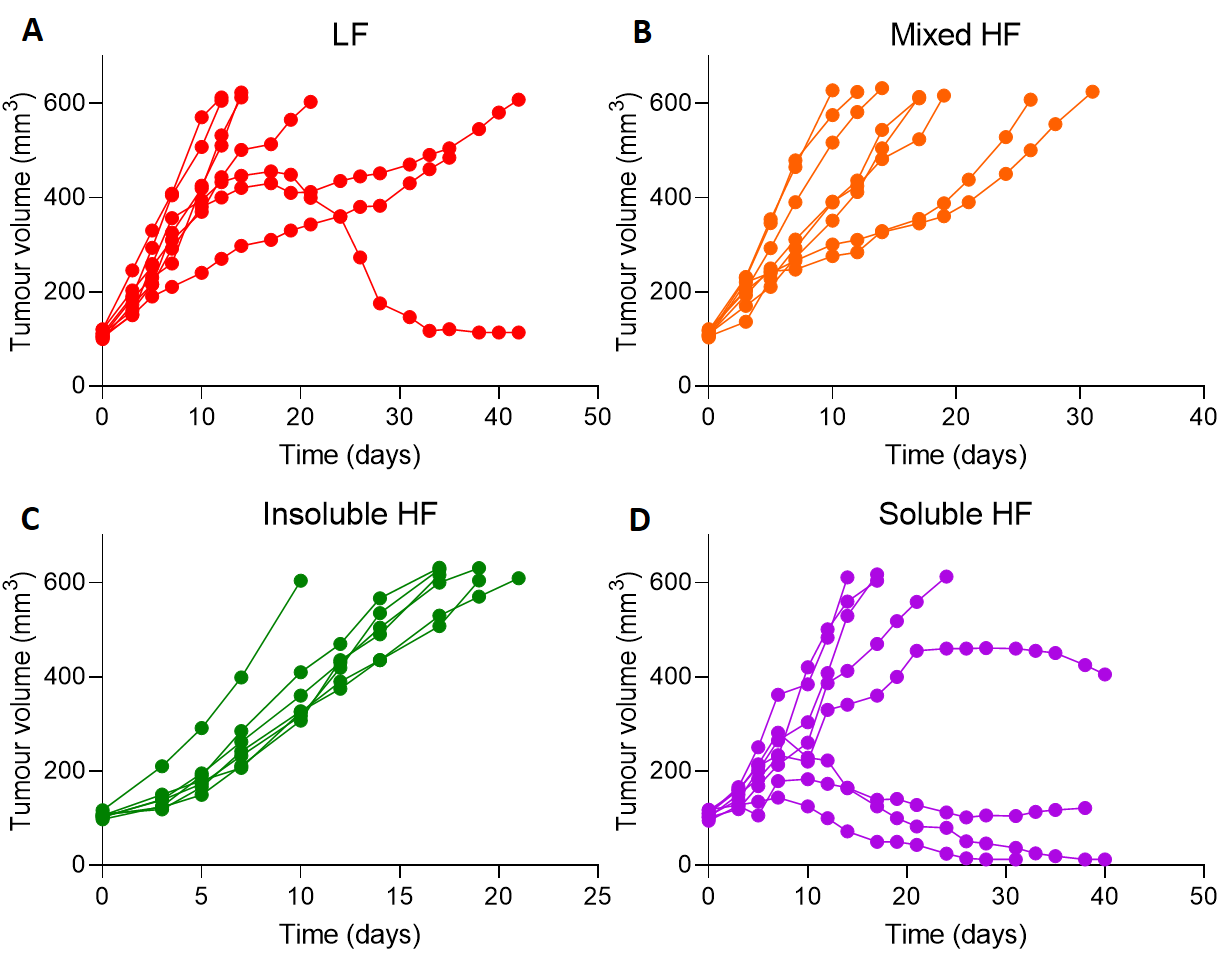
**

**Figure S4. Individual mouse tumour growth curves.** Tumour growth in RT112 flank xenografts irradiated with 6 Gy IR, in mice fed low fibre (A), high mixed fibre (B), high insoluble fibre (C) and high soluble fibre diets (D) (n = 8 for each group).

**Figure S5. Cell survival analysis of RT112 bladder tumour cells treated with SCFAs and bacterial supernatants.** (A) Inhibition of cell viability of RT112 cells single SCFA and combined SCFAs mixture in a time-dependent manner (N=3). The combined SCFAs denote the mixtures of 10 mM butyrate, 10 mM propionate, 10 mM butyrate for the left-hand graph and the mixtures of 10 mM butyrate, 5 mM propionate, 1.7 mM butyrate for the right-hand graph. (B) Reduced cell survival of RT112 cells by bacterial supernatants at day 3 (N=1). *BA*+*LP* denotes the cross-feeding of *B. acidifaciens* and *L. plantarum*, while *Bif*+*LP* denotes the cross-feeding of *Bifidobacterium* and *L. plantarum.* *P<0.05; **P<0.01; ***P<0.001.

**
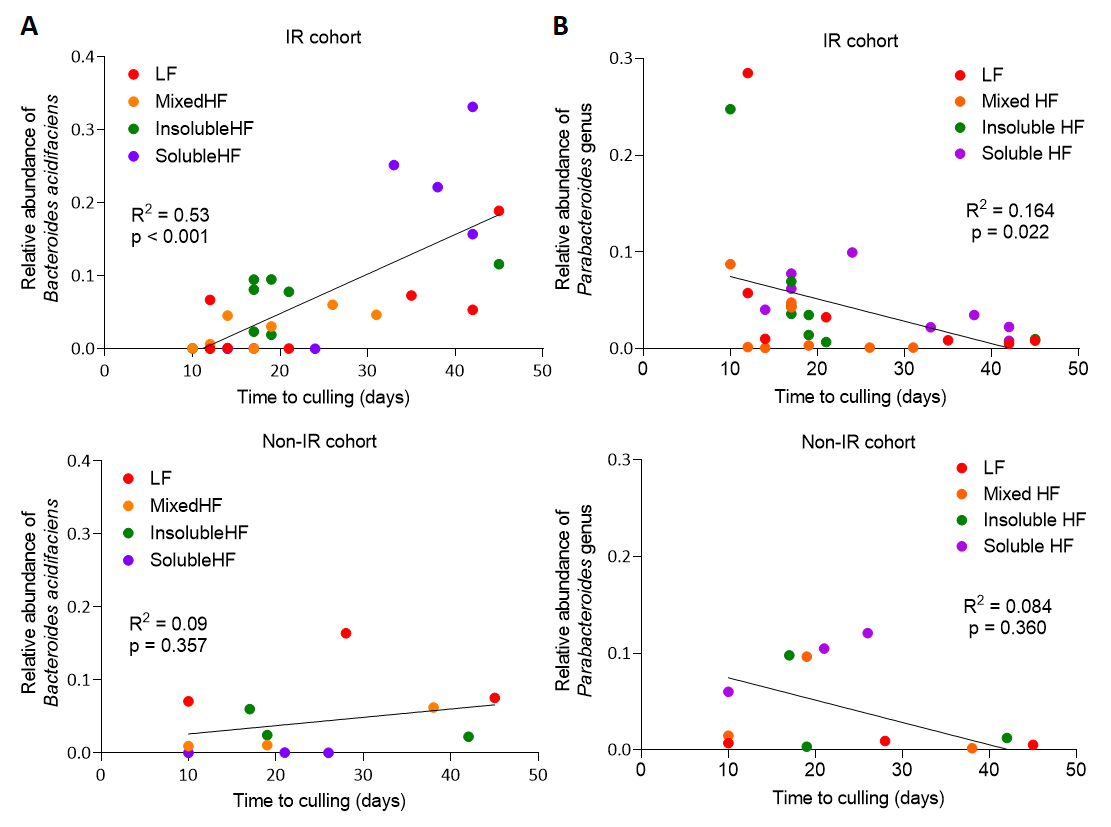
**

**Figure S6. Correlation of time to culling with *B. acidifaciens* or *Parabacteroides* genus abundance different groups.** (A) Correlation between time of culling versus (A) *B. acidifaciens* abundance (IR cohort, R^2^ = 0.53, p < 0.001; Non-IR cohort, R^2^ = 0.09, P = 0.357) or *Parabacteroides* genus abundance (IR cohort, R^2^ = 0.164, P = 0.022; Non-IR cohort, R^2^ = 0.084, P = 0.360) in the gut microbiome.

**
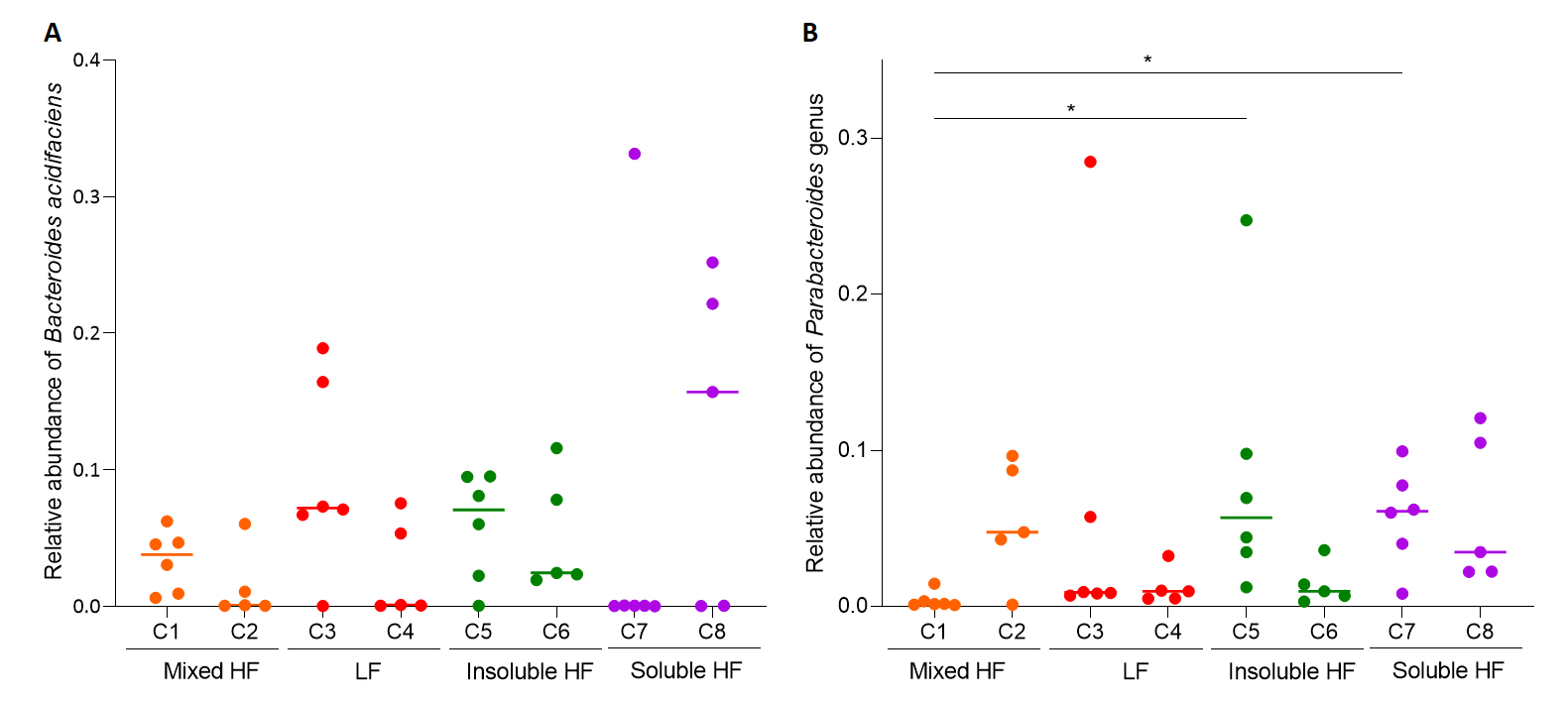
**

**Figure S7. Effect of cage location of mice on relative abundance of *B. acidifaciens* and *Parabacteroides* genus.** Comparison of (A) *B. acidifaciens* (p=0.200) or (B) *Parabacteroides* genus (p=0.005) abundance among mice from cage 1 (C1) to cage 8 (C8) by Kruskal-Wallis test.

Table S1. Rodent diets used in the study with varying levels of cellulose or inulin per 4000 kcal.

|  | Low fibre | |  | High insoluble fibre | | High soluble fibre | | High mixed fibre | |
| --- | --- | --- | --- | --- | --- | --- | --- | --- | --- |
|  | 2 gm Cellulose/3850kcal | |  | 100 gm Cellulose/3850kcal | | 100 gm Inulin/3850kcal | | 50 gm Cellulose  + 50 gm Inulin/3850kcal | |
|  | gm% | kcal% |  | gm% | kcal% | gm% | kcal% | gm% | kcal% |
| Protein | 21 | 20 |  | 19 | 20 | 20 | 20 | 20 | 20 |
| Carbohydrate | 67 | 64 |  | 61 | 64 | 59 | 60 | 60 | 62 |
| Fat | 7 | 16 |  | 7 | 16 | 7 | 16 | 7 | 16 |
| Total |  | 100 |  |  | 100 |  | 96 |  | 96 |
| kcal/gm | 4.20 |  |  | 3.81 |  | 3.95 |  | 3.88 |  |
|  |  |  |  |  |  |  |  |  |  |
| **Ingredient** | **gm** | **kcal** |  | **gm** | **kcal** | **gm** | **kcal** | **gm** | **kcal** |
| Casein | 200 | 800 |  | 200 | 800 | 200 | 800 | 200 | 800 |
| L-Cystein | 3 | 12 |  | 3 | 12 | 3 | 12 | 3 | 12 |
|  |  |  |  |  |  |  |  |  |  |
| Corn starch | 397.486 | 1590 |  | 397.486 | 1590 | 359.986 | 1440 | 378.736 | 1515 |
| Maltodextrin 10 | 132 | 528 |  | 132 | 528 | 132 | 528 | 132 | 528 |
| Sucrose | 100 | 400 |  | 100 | 400 | 100 | 400 | 100 | 400 |
|  |  |  |  |  |  |  |  |  |  |
| Cellulose, BM200 | 2 | 0 |  | 100 | 0 | 0 | 0 | 50 | 0 |
| Inulin | 0 | 0 |  | 0 | 0 | 100 | 0 | 50 | 75 |
|  |  |  |  |  |  |  |  |  |  |
| Soybean oil | 70 | 630 |  | 70 | 630 | 70 | 630 | 70 | 630 |
| t-Butylhydroquinone | 0.014 | 0 |  | 0.014 | 0 | 0.014 | 0 | 0.014 | 0 |
|  |  |  |  |  |  |  |  |  |  |
| Mineral mix S10022G | 35 | 0 |  | 35 | 0 | 35 | 0 | 35 | 0 |
|  |  |  |  |  |  |  |  |  |  |
| Vitamin mix V10037 | 10 | 40 |  | 10 | 40 | 10 | 40 | 10 | 40 |
| Choline bitartrate | 2.5 | 0 |  | 2.5 | 0 | 2.5 | 0 | 2.5 | 0 |
|  |  |  |  |  |  |  |  |  |  |
| **Total** | **952** | **4000** |  | **1050** | **4000** | **1012.5** | **4000** | **1031.25** | **4000** |
|  |  |  |  |  |  |  |  |  |  |
| Total Cellulose (gm/kg diet) | 2.1 |  |  | 95.2 |  | 0 |  | 48.5 |  |
| Inulin (gm/kg diet) | 0 |  |  | 0 |  | 98.8 |  | 48.5 |  |

Table S2. Details mouse diets, cages, *B. acidifaciens* relative abundance and time of culling

| Diet | **Low fibre** | | | | | | | | | | |
| --- | --- | --- | --- | --- | --- | --- | --- | --- | --- | --- | --- |
| Cage | 3 | | | | | | 4 | | | | |
| ID | C3.3 | C3.1 | C3.10 | C3.30 | [C3.NM](http://c3.nm/) | C3.4 | C4.10 | C4.1 | [C4.NM](http://c4.nm/) | C4.30 | C4.3 |
| IR/Non-IR | Non-IR | IR | IR | IR | IR | Non-IR | Non-IR | IR | IR | IR | IR |
| *B.acidifaciens* abundance | 0.164 | 0.000 | 0.067 | 0.189 | 0.073 | 0.071 | 0.075 | 0.000 | 0.053 | 0.001 | 0.001 |
| *Parabacteroides* genus abundance | 0.009 | 0.285 | 0.057 | 0.008 | 0.009 | 0.007 | 0.005 | 0.032 | 0.005 | 0.010 | 0.010 |
| Time for tumour to treble in volume | 17 | 6 | 6 | 6 | 14 | 5 | - | 9 | 8 | 7 | 8 |
| Time to culling (days) | 28 | 12 | 12 | 45 | 35 | 10 | 45 | 21 | 42 | 14 | 14 |
|  |  |  |  |  |  |  |  |  |  |  |  |
| Diet | **Mixed high fibre** | | | | | | | | | | |
| Cage | 1 | | | | | | 2 | | | | |
| ID | [C1.NM](http://c1.nm/) | C1.10 | C1.3 | C1.1 | C1.30 | C1.4 | C2.10 | [C2.NM](http://c2.nm/) | C2.3 | C2.30 | C2.1 |
| IR/Non-IR | IR | IR | IR | Non-IR | Non-IR | IR | IR | IR | IR | Non-IR | IR |
| *B.acidifaciens* abundance | 0.045 | 0.030 | 0.047 | 0.009 | 0.062 | 0.006 | 0.001 | 0.000 | 0.000 | 0.011 | 0.060 |
| *Parabacteroides* genus abundance | 0.001 | 0.003 | 0.001 | 0.015 | 0.002 | 0.002 | 0.087 | 0.048 | 0.043 | 0.096 | 0.001 |
| Time for tumour to treble in volume | 6 | 7 | 10 | 4 | - | 5 | 5 | 9 | 7 | 7 | 14 |
| Time to culling (days) | 14 | 19 | 31 | 10 | 38 | 12 | 10 | 17 | 17 | 19 | 26 |
|  |  |  |  |  |  |  |  |  |  |  |  |
| Diet | **Insoluble high fibre** | | | | | | | | | | |
| Cage | 5 | | | | | | 6 | | | | |
| ID | C5.3 | C5.30 | C5.1 | C5.10 | [C5.NM](http://c5.nm/) | C5.4 | [C6.NM](http://c6.nm/) | C6.1 | C6.3 | C6.30 | C6.10 |
| IR/Non-IR | Non-IR | IR | Non-IR | IR | IR | IR | IR | IR | IR | IR | Non-IR |
| *B.acidifaciens* abundance | 0.060 | 0.081 | 0.022 | 0.095 | 0.001 | 0.095 | 0.019 | 0.023 | 0.078 | 0.116 | 0.024 |
| *Parabacteroides* genus abundance | 0.098 | 0.069 | 0.012 | 0.035 | 0.248 | 0.044 | 0.014 | 0.036 | 0.007 | 0.010 | 0.003 |
| Time for tumour to treble in volume | 6 | 8 | 20 | 8 | 6 | 10 | 10 | 10 | 10 |  | 11 |
| Time to culling (days) | 17 | 17 | 42 | 19 | 10 | 17 | 19 | 17 | 21 | - | 19 |
|  |  |  |  |  |  |  |  |  |  |  |  |
| Diet | **Soluble high fibre** | | | | | | | | | | |
| Cage | 7 | | | | | | 8 | | | | |
| ID | C7.1 | C7.10 | C7.3 | [C7.NM](http://c7.nm/) | C7.30 | C7.4 | [C8.NM](http://c8.nm/) | C8.1 | C8.10 | C8.3 | C8.30 |
| IR/Non-IR | Non-IR | IR | IR | IR | IR | IR | IR | Non-IR | IR | IR | Non-IR |
| *B.acidifaciens* abundance | 0.000 | 0.000 | 0.000 | 0.001 | 0.331 | 0.000 | 0.221 | 0.000 | 0.252 | 0.157 | 0.000 |
| *Parabacteroides* genus abundance | 0.060 | 0.078 | 0.099 | 0.062 | 0.008 | 0.040 | 0.035 | 0.105 | 0.022 | 0.022 | 0.121 |
| Time for tumour to treble in volume | 6 | 10 | 11 | 9 | 12 | 7 | - | 7 | - | - | 12 |
| Time to culling (days) | 10 | 17 | 24 | 17 | 42 | 14 | 38 | 21 | 33 | 42 | 26 |
